# Validation of thoracic impedance cardiography by echocardiography in healthy late pregnancy

**DOI:** 10.1186/s12884-015-0504-5

**Published:** 2015-03-28

**Authors:** Jordan PR McIntyre, Kevin M Ellyett, Edwin A Mitchell, Gina M Quill, John MD Thompson, Alistair W Stewart, Robert N Doughty, Peter R Stone, the Maternal Sleep in Pregnancy Study Group

**Affiliations:** Department of Obstetrics and Gynaecology, Faculty of Medical and Health Sciences, University of Auckland, Level 12, Support Building, Auckland City Hospital, Private Bag 92019 Auckland, New Zealand; New Zealand Respiratory and Sleep Institute, Auckland, New Zealand; Respiratory Measurement Laboratory, Auckland District Health Board, Auckland, New Zealand; Department of Paediatrics, University of Auckland, Auckland, New Zealand; Department of Medicine, University of Auckland, Auckland, New Zealand; Department of Epidemiology and Biostatistics, School of Population Health, University of Auckland, Auckland, New Zealand

**Keywords:** Thoracic impedance cardiography, Pregnancy, Echocardiography, Stroke volume, Cardiac output, Non-invasive assessment

## Abstract

**Background:**

Assessment of stroke volume (SV) is often necessary in clinical and research settings. The clinically established method for SV assessment in pregnancy is echocardiography, but given its limitations, it is not always an appropriate measurement tool. Thoracic impedance cardiography (ICG) allows continuous, non-invasive SV assessment. However, SV determination relies on assumptions regarding the thoracic shape that may mean the algorithm is not valid in pregnancy. The available data regarding the validity of ICG against an established reference standard using modern SV algorithms are both limited and conflicting. We aimed to test the validity of ICG in a clinically realistic setting in late pregnancy using echocardiography.

**Methods:**

Twenty-nine women in late pregnancy underwent standard echocardiography assessments with simultaneous ICG measurement. Agreement between devices was tested using Bland-Altman analysis.

**Results:**

Bland-Altman analysis of the relationship between ICG and echocardiography demonstrated that the 95% limits of agreement exceeded acceptable or expected ranges. Measures of maternal and fetal anthropometry do not account for the lack of agreement.

**Conclusions:**

Absolute values of SV as determined by ICG are not valid in pregnancy. Further work is required to examine the ability of ICG to assess relative changes in maternal haemodynamics in late pregnancy.

## Background

The assessment and monitoring of stroke volume (SV) and cardiac output (CO) is often useful in research and for patient care. The currently established methods of SV measurement have the disadvantage of being invasive and carry a degree of risk to the participant (e.g. Fick principle and thermodilution), or are minimally invasive, but disruptive (e.g. echocardiography). In addition to requiring specialist personnel, such methods are time-consuming and continuous assessment is not always possible. The use of invasive or disruptive methods in assessment of healthy pregnant participants either presents unacceptable risk when not medically indicated, or is not practical, especially if the intent is to monitor over long periods of time, such as during sleep.

Echocardiography is clinically well established and provides a detailed and accurate assessment of cardiac structure and function in pregnancy. However, echocardiography has its limitations. Results are highly dependent on the skill of the echocardiographer [1], and in pregnancy can be limited by patient position. From a research perspective, echocardiography does not provide a continuous assessment of cardiac function over more than a few minutes and cannot be used in sleep studies. Consequently, a non-invasive method that permits continuous measurement, with less operator-dependency that can be used in ambulatory settings would be advantageous.

Thoracic impedance cardiography (ICG) may overcome some of the disadvantages of the other methods. ICG is a method of non-invasively estimating SV and assessing haemodynamic status via ECG surface electrodes, using complex algorithms to convert changes in thoracic impedance into volume changes. ICG has the potential to provide continuous (beat-to-beat) data which is less operator-dependent and (potentially) less influenced by patient factors such as body mass index (BMI). However, the algorithms used by ICG to calculate SV make assumptions regarding the thoracic dimensions and shape, which it assumes to be a truncated cone [2]. This truncated cone model, and the assumption that all patients have this thoracic shape, does not account for the differing anthropometric profile contributions to any increase in BMI (and therefore ignoring how it differs in men and pregnant women). Smith [3] highlighted many of the flaws to the assumptions made by ICG in an older, but still very relevant, letter to the editor, including the effect on impedance of the body size and shape, resistivity of blood and regional blood flow. Clearly, these are important with the greatly differing shape and distribution of blood flow of the pregnant body. The published data on ICG reliability and validity are inconclusive in a non-pregnant population, and there is a paucity of published data in a healthy late gestation pregnant population. The few available studies on ICG performance in pregnancy are inconsistent in their conclusions [4-9], in part possibly due to most being reports based on older equipment and algorithms, which have since been revised. As a result, further validation of ICG in pregnancy is required.

We aimed to examine the agreement between ICG and echocardiography in a cohort of healthy women in late pregnancy.

## Methods

### Participants

Healthy women aged ≥ 18 years with a normal singleton pregnancy, late in the third trimester, were recruited from low risk midwifery care. Exclusion criteria included: smoking or alcohol use, any medical or obstetric complications (e.g. intrauterine growth restriction, preeclampsia, any known cardiovascular, respiratory or renal disorders, all forms of diabetes), not regularly attending scheduled obstetric appointments, orthopaedic or musculoskeletal conditions which would make adopting different maternal positions difficult, multiple pregnancy, and extremes of body habitus (i.e. height <120 cm or >230 cm, weight <30 kg or >155 kg, which have been reported to affect the accuracy of ICG [10]). All participants were assessed by the study obstetrician on the day of this study to ensure there were no pregnancy abnormalities and an obstetric ultrasound was performed to check fetal welfare. Birth outcome data were collected to confirm the health status of the fetus-neonate.

### Experimental design/protocol

All studies were performed under standardized conditions in the afternoon. Participants were asked to abstain from all caffeine containing foods, chocolate and strenuous exercise on the day of study. Upon arrival, height and weight were measured to determine current BMI and body surface area (BSA). Self-reported pre-pregnancy weight was obtained to determine pre-pregnancy BMI. Participants then rested for 30 minutes prior to the assessment during which time the electrodes were placed.

### Echocardiography

Limited two-dimensional transthoracic echocardiograms were performed by one qualified cardiac sonographer using a Philips iE33 ultrasound machine (Bothell, WA, USA). Patients were scanned in the left lateral decubitus position. Standard transthoracic echocardiographic views and measurements were acquired as per the recommendations of the American Society of Echocardiography guidelines [11,12], ensuring normality of cardiac anatomy and allowing for the measurement of left ventricular outflow tract velocity time integral (LVOT_VTI_) and diameter (LVOT_d_) to calculate SV and CO.

During a period of stable cardiac rhythm, a Doppler sample volume was placed within 1 cm of the aortic valve in the LVOT and approximately 10 consecutive beats were recorded in frozen screen format. This was performed three times during the examination: beginning (3.1 ± 1.3 minutes), mid-point (10.7 ± 2.3 minutes), and end (17.5 ± 2.8 minutes) (these are termed epochs 1, 2 and 3, respectively in the results). CO was derived from real-time measurements of three averaged consecutive cardiac cycles at each of the three time points.

### Impedance cardiography

ICG data was collected continuously for the duration of the echocardiography assessment. The CardioScreen 1000® (Medis Medizinische Messtechnik GmbH, Ilmenau, Germany) was attached to participants according to manufacturer’s instructions using disposable adhesive dual-surface electrodes in the lateral spot configuration, after preparation with abrasive gel (Nuprep® Skin Prep Gel, DO Weaver and Co., Aurora, CO, USA).

To allow direct comparison and synchronisation with echocardiography, electronic markers were placed in the ICG data at the first of each three averaged echocardiography SV values, and one minute of ICG values were averaged to give the SV value for that time point (30 seconds either side of the echocardiography marker). As the echocardiography computer only gave a time at which the image was captured and the mean HR for that captured image, and not an exact time for each RR interval, it was not possible to compare the exact same cardiac cycles between devices. Thirty seconds of ICG data either side of echocardiography was judged to be the best alternative, minimising any significant effects of any potential outlier SV values if using a shorter time period. The device used its own ECG signal to determine HR and calculate CO from SV. SV was determined using the in-built propriety PASA algorithm (Physiological Adaptive Signal Analysis) [13], a modification of Sramek-Bernstein algorithm [2].

The operators of the two different devices were blinded to the values produced by the other during data collection and retrospective analysis.

### Data management

The relationship between ICG and echocardiography at each epoch was analysed separately. Six velocity spectrums at each epoch were re-measured offline in a subset of 20 participants to report intra-observer variability. This was also used to test if the number of averaged cardiac cycles, or whether “online” or “offline” analysis of echocardiography waveforms (i.e. during or after the echocardiogram), influenced echocardiography agreement with ICG. The echocardiography coefficient of variation (CoV) across the three epochs was compared between averaging methods: mean of three beats per epoch online vs. mean of three per epoch offline vs. mean of six per epoch offline. For ICG, CoV was calculated using the 60 seconds of data at each of the three epochs.

An analysis of variance (ANOVA) was used to test if measures of HR, SV and CO changed significantly between epochs for each device. The agreement between echocardiography and ICG values for SV and CO was assessed for the full dataset using Bland-Altman analysis [14], with each epoch assessed separately, so as to not violate the assumptions of independence between samples [15]. In addition to absolute difference between devices, percentage error for each sample was calculated as the absolute difference between echocardiography and ICG divided by the echocardiography value, times 100.

The CoV across the three epochs was calculated from the individual change in values between epochs and used to compare the overall variance between devices. Pearson correlation coefficients examined any relationship that maternal and fetal anatomical variables might have with absolute SV and CO values and the difference in ICG and echocardiography values.

We have used one outcome (CO) to calculate a sample size of 30 subjects. This assumes a SD of CO of 2.0 L/min. The cross over design makes it likely that there will be a correlation between observations and if we assume this to be 0.5, the difference detectable with 80% power at the 5% level of significance is 1.0 L/min, which is of potential clinical significance.

The Regional Human Ethics Committee approved the study protocol (NTX/11/09/084), and all participants provided written informed consent.

## Results

Twenty-nine participants completed this study. Twenty-one (72%) were Caucasian. Mean age was 30.5 ± 5.5 years, BMI: 28.8 ± 5.3 kg · m^−2^, and BSA: 1.9 ± 0.2 m^2^. Pre-pregnancy BMI (23.4 ± 4.2 kg · m^−2^) was comparable to that of the local obstetric population [16]. Twenty (69%) were primigravid and 23 (79%) nulliparous. Mean gestation was 37 weeks, estimated fetal weight 3062 ± 715 g, and deepest pool of amniotic fluid 5.5 ± 1.7 cm. Birth weight data was available to the researchers on 27 of the new-borns. Gestation at birth was 40 ± 1 weeks (n = 29) and birth weight 3469 ± 417 g (n = 27). All reported live births, free of congenital abnormalities. The echocardiogram assessment lasted 19.6 ± 4.1 minutes on average.

### Effect of number of cardiac cycles averaged on variability of echocardiography

The CoVs for the three averaging methods were 6.7% for the “online” measures, 7.5% when three offline measures were averaged per epoch, and 10.4% for six offline measures at each epoch. This indicated that echocardiography values have the lowest variation when measured “online”, that is during the assessment, compared with tracing the velocity spectrums retrospectively. Therefore, the mean of three consecutive beats (online) was used in all comparisons with ICG.

### Echocardiographic results from healthy third trimester pregnant women

All women had normal cardiac structure and function (consistent with pregnancy) on the echocardiogram. The echocardiographic measures of LVOT, HR, SV and CO are shown in Table 1.

**Table 1:** **Echo results, n = 29**

	**Mean ± SD**
LVOTd (cm)	2.1 ± 0.2
LVOT VTI (cm)	22.8 ± 3.9
LVOT V_max_ (cm.s^−1^)	116.0 ± 16.5
HR (bpm)	71.8 ± 11.7
SV (mL)	79.4 ± 19.7
CO (L.min^−1^)	5.6 ± 1.4

### Echocardiographic assessment of participants and validation of impedance cardiography

**Figure 1:**
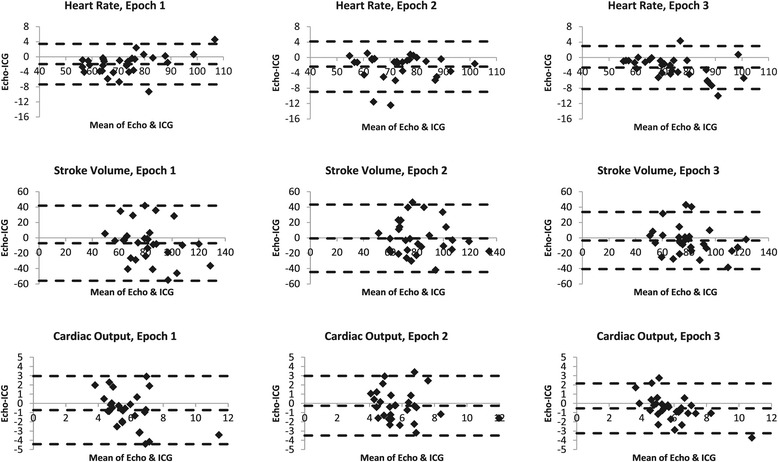
**Bland-Altman plots; Echocardiography vs. Impedance Cardiography.** Broken lines indicate mean ± 1.96 SD. HR, SV and CO are in beats per minute, mL, and L.min^−1^, respectively.

The mean and SD of HR, SV and CO for the echo and ICG are presented in Table 2. Within-device differences in HR, SV and CO across the three epochs only reached statistical significance for echocardiography-derived CO between epochs one and two (mean difference: −0.27 L.min^−1^, p = 0.04, 95% CI: −0.53 to −0.007). The difference between echocardiography and ICG is plotted against the average of the two values for each participant in the Bland-Altman plots in Figure 1, with horizontal lines indicating the mean difference ± 95% CI. The mean difference (lower and upper limits of 95% CI) for the three epochs was HR: −2.0 (−7.3, 3.4), −2.4 (−8.9, 4.1), −2.6 (−8.2, 3.0) bpm; SV: −7.0 (−55.8, 41.8), −0.6 (−44.5, 43.3), −3.5 (−40.6, 33.5) mL; CO: −0.7 (−4.4, 3.0), −0.3 (−3.5, 3.0), −0.5 (−3.2, 2.2) L.min^−1^. This equated to a mean percentage error for the three epochs of HR: 3.7 (−2.5, 9.9), 3.8 (−6.2, 13.8), 3.9 (−1.9, 9.7); SV: 26.2 (−21.4, 73.8), 23.1 (−8.6, 54.8), 18.7 (−13.7, 51.1); CO: 29.2 (−19.3, 77.6), 24.2 (−10.5, 58.9), 20.1 (−14.4, 54.6). The mean HR for the 60s of ICG data had excellent correlation with the echocardiography HR, given as a mean value of all beats in the captured image (R = 0.98, 0.96, 0.98 for epochs 1–3, respectively). There was no correlation between maternal BMI, maternal BSA, estimated fetal weight or deepest pool of amniotic fluid and the differences between ICG and echocardiography SV or CO values at any epoch (r value range: −0.33-0.17; p = 0.076-0.895). BMI demonstrated a significant but marginal relationship with absolute CO in all epochs with both devices, with absolute HR in epochs one and two, but no significant correlations with SV. BSA was significantly related with both SV and CO in all epochs for both devices (r = 0.45-0.70; p = 0.000-0.014), except for ICG-SV in epoch two (r = 0.35; p = 0.061). Neither estimated fetal weight nor deepest pool of amniotic fluid correlated with CO or SV.

**Table 2:** **Echo vs. ICG at epochs 1, 2 and 3**

	**Echo**	**ICG**
HR (bpm)		
1	71.4 ± 13.0	73.3 ± 11.9
2	71.8 ± 11.6	74.2 ± 11.7
3	72.5 ± 11.7	75.1 ± 13.0
SV (mL)		
1	78.8 ± 19.1	85.8 ± 24.9
2	81.3 ± 21.4	81.9 ± 23.5
3	78.1 ± 18.5	81.6 ± 22.8
CO (L.min^−1^)		
1	5.5 ± 1.3	6.2 ± 2.0
2	5.8 ± 1.7	6.0 ± 1.9
3	5.6 ± 1.2	6.1 ± 1.9

Mean ± SD (n = 29).

The CoVs for the echocardiograms and the ICG based in individual change across the three epochs are presented in Table 3. For both devices, the CoV for SV and CO is comparable within devices. However, the CoV for SV and CO values as determined by ICG are markedly higher than the corresponding echocardiography values.

**Table 3:** **Coefficient of variations for Echo and ICG, n = 29**

	**Echo**	**ICG**
HR	0.3 ± 1.4% (−2.4%, 3.0%)	7.7 ± 4.1% (−0.3%, 15.8%)
SV	6.7 ± 2.6% (1.6%, 11.9%)	19.3 ± 9.3% (1.2%, 37.5%)
CO	6.5 ± 2.5% (1.7%, 11.4%)	20.7 ± 9.4% (2.2%, 39.1%)

Mean CoV ± SD (95% Confidence Intervals).

## Discussion

In the current study, it was found that ICG and echocardiography have poor agreement in terms of SV and hence CO determination. This study was designed to reflect a clinical environment, such that it could reasonably be expected to apply this protocol in the clinical assessment of pregnant women. Conditions were standardized as far as was clinically reasonable, whilst permitting optimal measurement of the cardiovascular parameters being compared. The aim was to compare the ICG with an established non-invasive method. Echocardiography meets that criteria in both non-pregnant [17] and pregnant populations [18].

Our LVOT_VTI_, HR, SV and CO values were comparable to published echocardiography values in similar gestation women [19,20]. ICG-derived mean values for SV and CO in the current paper were also comparable to those previously published in similar gestation women [21]. At 6.5 ± 2.5%, the echocardiography CoV in the current study is lower than previously reported 8.8% for cardiac output [22].

There was excellent correlation between echocardiography and ICG derived HR, and clinically insignificant differences between the devices in the Bland-Altman analysis. Therefore, we can be confident that the HR component of ICG is stable and accurate across the two devices, and any discrepancies in CO can be attributed to SV. The non-perfect correlation between ICG and echocardiography-derived HR values (R = 0.96-0.98) can be attributed to the different time periods (three consecutive cardiac cycles by echocardiography compared with a continuous measure of 60 seconds of beat-beat data from ICG). Ideally, the same three cardiac cycles would be compared between the two devices, rather than three beats versus 60 seconds of data. However, the echocardiography gave an averaged, rather than instantaneous, heart rate, making such precise matches with ICG impossible. As we were unable to synchronise beats between echocardiography and ICG, a one minute mean of ICG appeared to be the next best option. Selecting fewer beats of ICG data would introduce a potential for greater variability due to respiration if indeed different beats were selected to those analysed by echocardiography. There was large variability in ICG values compared with echocardiography (Table 3). This may be due to the greater number of beats averaged in each ICG sample compared with echocardiography (60s vs. three beats), or it could imply that ICG is less stable and may account for a greater proportion of the error between the two methods.

A much cited paper by Critchley and Critchley [23] rightly states that any evaluation of CO measurement devices should take into consideration the precision of the reference method. They state that a previous review had rejected CO measurement validation studies based on percentage error that should have been accepted when their adjusted limits of agreement of 30% were used. Of note, one of the studies Critchley and Critchley [23] re-categorised as satisfactory was validating echocardiography against thermodilution, adding validity to the reference standard used in the current study. A previous study [24] on the validity of different ultrasound methods stated that echocardiography had a percentage error of 10% based on earlier published data. If we were to assume the same 10% error in our reference measure, using the best percentage error (percent limits of agreement) in the third epoch of approximately 20% (14-55%), our results certainly fall outside of those deemed acceptable in the error-gram of Critchley and Critchley [23]. Even using the more conservative 20% precision (as used for thermodilution), our limits of agreement still fall outside of the 30% limits of agreement recommended by of Critchley and Critchley [23].

Burlingame et al. [4] compared echocardiography with ICG in 28 women in late pregnancy (34.0 ± 1.2 weeks gestation) when in the left lateral position. They used the BioZ device with the ZMARC algorithm, which the Medis ICG device and PASA algorithm are branded as in some countries. They reported comparable values to the current study, with SV of 74 ± 21 and 84 ± 24 mL, and CO of 5.8 ± 1.6 and 6.4 ± 1.9 L.min^−1^, for echocardiography and ICG respectively. However, the mean difference (confidence intervals) between the two devices at 15.1 mL (−29.1, 59.3) and 1.0 L.min^−1^ (−2.9, 4.9) for SV and CO, respectively, was higher than the current study, which ranged from −7.0 to −0.6 mL and −0.7 to −0.3 L.min^−1^. Interestingly, Burlingame et al. [4] concluded that ICG was acceptable, even with these wide confidence intervals.

Burlingame et al. [4] also reported that ICG was able to detect subtle changes in SV when changing from supine to seated. They did not however measure SV in the supine position using the reference method, echocardiography, but rather just noted the changes in ICG values with position change. Therefore, whilst it can be said that ICG can detect a change in SV with position, the accuracy in the magnitude of change was not demonstrated. The ability to detect changes in SV with maternal position is questionable, given that it has been reported that ICG is only valid in lateral positions [9]. Using the ICG device NICCOMO (with the same manufacturers and SV algorithm as the device in the current study), Tomsin et al. [25] cited moderate to high inter- and intra-session reliability (Pearson’s Correlation Coefficient) of various measures in uncomplicated third trimester pregnancies in both supine and standing positions. They did not report correlations between the different positions, nor with another reference device. Moertl et al. [17] investigated the ability of ICG to monitor changes in SV in six assessments over the course of pregnancy. They assessed individual components of a modified version of the older Kubicek SV algorithm [26] within a narrow range of RR-intervals in each of the participants’ repeated assessments, as well as SV and CO. They concluded that implausible and unreliable results values were obtained, and that ICG appeared unsuitable for monitoring changes in CO over pregnancy. One previous study reported that ICG-measured changes in SV with position change in late pregnancy were of a magnitude with thermodilution [5]. This was however an older study that used a different electrode configuration and the first generation Kubicek algorithm [26].

Consideration of these unfavourable reports, along with our own findings, does question the value of ICG in late pregnancy. ICG produced SV and CO values that were both greater and less than the corresponding echocardiography values, resulting in large limits of agreement (Figure 1). This indicates that absolute values cannot be used. That the differences between ICG and echocardiography did not correlate with maternal BMI and BSA, estimated fetal weight (a surrogate for uterine size) or deepest pool of amniotic fluid, indicate that these variables cannot be held accountable for any differences in values between devices. The error is then likely attributable to some flaw of either the ICG device or its algorithm producing random error, or error of echocardiography, in this population.

The algorithms employed by ICG to calculate SV are dependent on assumptions regarding the shape and dimensions of the thorax. The Sramek-Bernstein algorithm [2] is one that many manufacturers incorporate into their devices, or at least use propriety modifications of, including the device used in this study. The Sramek-Bernstein algorithm introduced a correction factor to account for deviations from an ideal BMI of 24 kg · m^2^ and their effects on the volume of electrical participating tissue (*V*_*EPT*_) portion of the algorithm. It would not however account for the different body mass distribution accounting for the increases in BMI in pregnant women compared with males that it was tested in. Additionally, it has been pointed out that the correction factor does not account for possible effects of gender differences in anthropometry on resistance to current, and thus ICG outputs [27]. The differences in anatomy between men and pregnant woman of similar BMI, such as thoracic circumference and shape, and fat-lean mass ratio, may mean that it is not appropriate to use the standard SV algorithms in pregnancy. It has been observed that the outputs from ICG are influenced by the sizes and positions of the electrodes, the patient’s body shape and size, and “the resistivity of the blood and distribution of other resistivities within the body” [3]. Highlighted also was the non-uniformity of the current flow throughout the whole of the thorax, whereby due to the electrode configuration, outputs will be influenced greatly by small volume flow changes in the neck, but less so by large changes in the arms, for example. Finally, Smith [3] described how the concept of ICG is using a simple model to describe complex changes. This simple model does not account for the potential effects on impedance of uneven blood perfusion within the thorax and the differing blood flow between heart and rest of the body.

The changing thoracic anatomy, shape and fluid content with increasing gestation also mean that the algorithms are unlikely to be able to be used to assess differences in SV or CO at different gestations, consistent with the findings of Moertl et al. [17]. The individual anatomical variations in pregnancy would rule out any ability to compare values between individuals, even at similar gestations. An individual’s specific thoracic anatomy in pregnancy may even preclude comparison with population norms, such as those reported by Morris et al. [21]. A more likely utility of the ICG would be in investigation of the physiological effects of an acute intervention. This would depend on whether the difference between ICG and echocardiography we have observed in the current study is due to a systematic error of the ICG which remains constant over the course of the intervention, thus providing a reliable measure of relative change. The ability of ICG to accurately measure relative changes in maternal haemodynamics in late pregnancy would need to be demonstrated with a suitable reference measure. Using a device by the same manufacturers (and therefore same algorithm), ICG was reported to unreliably detect induced changes in SV compared with echocardiography in healthy non-pregnant participants [28]. In the current study, there was no physiological challenge that may induce changes in SV of a magnitude that would be able to test the ability of ICG to detect those changes during the simultaneous measurement of echocardiography as Fellahi et al. [28] did (and Secher et al. [26] using the older algorithm). One method could be to test the effect of maternal recumbent position change on SV as measured by both devices. However, it is worth considering that different maternal positions in late pregnancy can potentially cause changes in thoracic geometry, which may invalidate the algorithm, and potentially also change maternal haemodynamics of a magnitude undetectable by ICG. Additionally, echocardiography can only be used effectively in late pregnancy (or indeed in most other situations) in a left lateral position. Previous work reported that ICG in pregnancy has good agreement with echocardiography only in the left and right lateral positions, with poor agreement when the women were in supine, sitting, standing or “knee-chest” positions [9]. This presents argument for a) changing fluid distribution with differing positions influencing accuracy of SV algorithms, b) the inability of ICG to accurately detect absolute changes in SV, or c) a reduction of accuracy of the reference standard, echocardiography, in non-lateral positions. Although Fellahi et al. [28] employed a different protocol, including artificially-induced haemodynamic load challenges, this demonstrates that the poor performance of ICG, and this algorithm in particular, cannot be attributed wholly to the pregnant condition of our participants and would, in our opinion, remove the need for a non-pregnant control group in the current study. Given that Fellahi et al. [28] also employed echocardiography as their reference measure, error associated with echocardiography (in preference to ICG) however cannot be discarded. A previous review reported a significant effect (p = 0.03) of the reference method used on how valid the ICG data was deemed to be [29]. Indirect Fick studies had the highest correlation at r = 0.91, compared with dye dilution (r = 0.82) and echocardiography (r = 0.69). Of course, we cannot determine from these studies if the error can be attributed to ICG or the reference standard. However, in the current study we only assessed in the left lateral position, which is shown to give the best agreement between ICG and echocardiography [9], and the clinically accepted position for accurate echocardiographic data. Therefore, we can be confident that we minimised the error associated with both position and the reference standard measure. The utility of ICG to assess maternal physiology with position changes remains to be proven.

### Limitations

The assessment was standardised as much as possible whilst not impeding on the echocardiography assessment and in keeping it as realistic to clinical assessment conditions as possible. However, it must be acknowledged that the assessment was standardised firstly to the echocardiography, in that it was designed to reflect a normal echocardiography assessment of cardiovascular function, and secondly to have the most robust ICG outputs possible. Participants were encouraged not to talk during SV measurement (and during the corresponding 60s ICG sample periods) and were given no instructions regarding breathing. This ensured the best possible conditions, with spontaneous breathing only and minimal effects of a variable respiratory rate (i.e. due to occasional sighs etc.). Breathing may have been standardised further by paced breathing, but it was felt that this was artificial and not representative of the conditions that ICG would be employed in (i.e. during sleep), and it would have been difficult to find a uniform respiratory rate that suited all women. Variations in respiratory rate and volume (e.g. due to an individual sigh) is unlikely to have affected mean SV over the 60 second ICG sample period, and the echocardiographer ensured only “normal” breaths during echocardiography SV measurement. Additionally, as pointed out by Burlingame et al. [4], improvements have been made in ICG signal analysis software to minimise the effects of respiratory variations.

Employing echocardiography as a reference measure may also be seen as a limitation. However, echocardiography is a clinically accepted, safe and non-invasive method of cardiovascular assessment in pregnancy. Previous published data has demonstrated the clinical validity of echocardiography, even with greater variability demonstrated than in the current study [22].

Lastly, as highlighted above, the different sample periods is another potential limitation. One potential method to improve agreement between devices may be to measure the LVOT_VTI_ continuously using echocardiography for the whole 60 seconds of the ICG sample period and retrospectively tracing around all the velocity spectrums.

## Conclusion

In conclusion, SV and CO as measured by ICG and echocardiography in healthy late pregnancy demonstrates a moderate relationship at best. The between-device agreement as illustrated by the Bland-Altman plots was poor throughout the assessment. Maternal or fetal variations in body proportions or size do not appear to account for the lack of agreement. We cannot categorically attribute all the error to the ICG and we acknowledge that echocardiography may have some inherent error. However, given the poor agreement between ICG and this clinically accepted tool, we cannot endorse the use of ICG to assess SV in a healthy pregnant population. Our results do not conclusively prohibit the use of ICG in this population, but it is yet to be proven that it can be used in this setting. Further work must be done to examine the ability of ICG to assess relative changes in maternal haemodynamics in late pregnancy.

## Abbreviations

BMI: Body Mass Index
BSA: Body Surface Area
CO: Cardiac Output
CoV: Coefficient of Variation
ECG: Electrocardiogram
ICG: Thoracic Impedance Cardiography
LVOT: Left Ventricular Outflow Tract
LVOTd: LVOT diameter
LVOT_VTI_: LVOT Velocity Time Integral
SV: Stroke Volume
